# Therapeutic effect of Botulinum toxin-A in 88 patients with Trigeminal Neuralgia with 14-month follow-up

**DOI:** 10.1186/1129-2377-15-43

**Published:** 2014-06-22

**Authors:** Shuang Li, Ya-Jun Lian, Yuan Chen, Hai-Feng Zhang, Yun-Qing Ma, Cai-Hong He, Chuan-Jie Wu, Nan-Chang Xie, Ya-Ke Zheng, Yi Zhang

**Affiliations:** 1 Department of Neurology, the First Affiliated Hospital, Zhengzhou University, 1 Jianshe East R, Zhengzhou 450052, Henan Province, People’s Republic of China

**Keywords:** Trigeminal neuralgia, Botulinum toxin A, Long-term therapeutic effect, Subcutaneous injection

## Abstract

**Background:**

We investigated the long-term effects and safety of botulinum toxin-A (BTX-A) for treating trigeminal neuralgia (TN). We also studied long-term maintenance of this therapeutic effect.

**Methods:**

A visual analog scale (VAS) score, pain attack frequency per day, patient’s overall response to treatment and side effects during 14-month follow-up were evaluated in 88 patients with TN receiving BTX-A. The primary endpoints were pain severity (assessed by VAS) and pain attack frequency per day. The secondary endpoint was the patient’s overall response to treatment, assessed using the Patient Global Impression of Change. The influence of different doses (≤50, 50–100 and ≥100 U) on the therapeutic effect was evaluated.

**Results:**

Treatment was deemed “effective” within 1 month in 81 patients and at 2 months in 88 patients (100%). The shortest period of effective treatment was 3 months, and complete control of pain was observed in a maximum of 46 patients. The therapeutic effect decreased gradually after 3 months, and the prevalence of effective treatment at 14 months was 38.6%, with complete control of pain seen in 22 patients (25%). There was no significant difference in the prevalence of effective treatment between different dose groups at identical time points (p > 0.05). Three patients showed swelling at injection sites and 10 patients showed facial asymmetry, both of which disappeared spontaneously without special treatment.

**Conclusion:**

Local subcutaneous injection of BTX-A for TN treatment has considerable therapeutic effects lasting several months and is safe for this indication. At least one-quarter of patients maintained complete analgesia. The maintenance period of the therapeutic effect may be related to the reduction in the VAS score after the first injection of BTX-A.

## Background

Trigeminal neuralgia (TN) is characterized by sudden (usually unilateral), severe, brief, stabbing, recurrent pain in the distribution of one or more branches of the trigeminal nerve. TN is usually evoked by the stimulation of facial “trigger points”, such as teeth-brushing, face-washing, drinking, or shaving. Because the pain is long-term and severe, the quality of life of patients is severely affected [1].

Botulinum toxin A (BTX-A) is a type of exotoxin produced by *Clostridium botulinum*. It relaxes muscles through the release of acetylcholine at nerve–muscle junctions. Therefore, BTX-A is used widely in cosmetology and treating dysmyotonia [2]. BTX-A can also inhibit secretion from glands through inhibition of the release of acetylcholine from cholinergic nerve terminals [3]. It is used in the treatment of bromhidrosis, hyperhidrosis, hygrostomia, vasomotor rhinitis, and rhinorrhea.

Recently, the use of BTX-A for pain management has received considerable attention. In 2002, Micheli et al. were the first to report that BTX-A can relieve TN [4]. Subsequent reports also supported the beneficial effects of BTX-A in TN treatment [5,6]. The longest observation period of the therapeutic effect of BTX-A in TN is 6 months [7,8]. In a review focusing on BTX for the treatment of neuropathic pain, the longest observation period for BTX-A in TN was 4 months [9]. Reports focusing on longer observation periods or factors related to the maintenance of long-term effects are lacking. Here, we report the effect of BTX-A in the treatment of 88 TN patients with a follow-up of 14 months.

## Methods

### Ethical approval of the study protocol

The study protocol was approved by the Ethics Committee of Zhengzhou University (Zhengzhou, China). All patients provided written informed consent to participate in the study.

### Patient recruitment

We conducted an open study. Eighty-eight subjects with primary one-branch classical TN treated with BTX-A were recruited as outpatients or inpatients from the Neurology Department of the First Affiliated Hospital, Zhengzhou University from January 2009 to June 2012. The goal, procedure and safety aspects of the study were explained to each of the patients and Informed consent was included in the documents of each patient. The medications which were used to alleviate their pain were asked to remain unchanged during the course of the study. All patients showed symptom improvement after 8 weeks of treatment, and were followed up for 12 months. And for some patients who didn’t show symptom improvement within 2–4 weeks,we gave an additional dose, and plus the additional dose for the first dose as the treatment dose. The patients who showed improvement after 8 weeks of re-injection were followed up for 12 months too. Before hospital admission, all patients underwent laboratory examinations to exclude coagulation disorders and severe dysfunction of the heart, liver or kidneys. Detailed physical examinations as well as CT and MRI of the cranium were conducted to exclude secondary TN.

### Therapeutic regimens

BTX-A crystals (HengLi^®^; 100 U/bottle; Lanzhou Bioproduction Institute, Lanzhou, China) was diluted with 0.9% saline (2 mL) to 50 U/mL before use. The BTX-A solution was injected in the facial area and the trigger point of pain at a depth of 0.5 cm, 2.5-5 IU per point, separation of 15 mm and at 15–20 injection points. And for some patients the pain within one side of gum, the injection points were 4–10,and because the gingival mucosa is thinner than skin, the dosage were fewer than facial area in each point.

### Observation indices and evaluation of therapeutic effects

Information regarding pain (including the extent of pain, pain attack frequency,and side effects) was recorded in detail each day by patients before and after therapy. Clinic visits or telephone follow-up were undertaken by the same person every week for 2 months and every month thereafter. The patient’s overall response to treatment was followed up each month after therapy. The extent of pain was evaluated through a visual analog scale (VAS) with 11 points on a ruler of length 10 cm where 0 denoted “no pain” and 10 denoted “most severe pain”. Patients pointed to the representive point of pain on the panel without reading it but instead according to their feelings, and the corresponding pain scores were recorded. We adopted the classification that a reduction in the VAS score of <50% after treatment meant that treatment was classified as being “ineffective”, 50–70% as “effective”, >75% as “significantly effective” and “0” meant that pain was “completely controlled”. The total effective value was defined as the percentage of patients with a reduction in the VAS score of ≥50%. The patient’s overall response to treatment assessed using the Patient Global Impression of Change. PCIC is a single-item rating by patients of their improvement with treatment during the therapy on a 7-point scale that ranges from ‘very much improved’ to ‘very much worse’ with ‘no change’ as the mid-point.

### Side effects

Information regarding side effects (time, extent, duration, frequency, treatment, outcomes of treatment, and correlation with treatment) was recorded in detail.

### Statistical analyses

Data are the mean ± SD and were analyzed using SPSS ver20.0 (SPSS, Chicago, IL, USA). The effective value was analyzed using the χ^2^ test. p *<* 0.05 was considered significant.

## Results

### General information

The patient cohort comprised 38 males (43.2%) and 50 females (56.8%). The age range was 33–89 years (mean, 60.6 years; SD = 11.90). Disease progression was 14–150 months with mean of 52 months and SD of 50.17. All 88 patients showed symptom improvement within 2 weeks. The percentage of patients in whom treatment was effective at 2 months reached 100%.

### Distribution of pain area

The pain area was in the first branch of the trigeminal nerve in 19 cases, in the second branch in 42 cases, and in the third branch in 27 cases.

### Injection dose

Maximal and minimal doses were 170 U and 25 U, respectively. The dose was ≤50 U in 43 cases, 50–100 U in 32 cases, and ≥100 U in 13 cases.

### Therapeutic effect at different time points

Treatment was classified as being effective within 1 month in 81 cases, and was effective at 2 months in 88 cases (prevalence of effective treatment of 100%). All the patients who showed symptoms improvement significantly reduced pain attack frequency. TN was classified as being completely controlled at 3 months in 46 cases. The number of cases in whom treatment was classified as being effective at 3 months decreased to 76, and the number of cases with a reduction in the VAS score of 50–75% decreased from 30 to 17 at 2 months. After 3 months, the effect of BTX-A decreased gradually, with a prevalence of effective treatment of 38.6% at 14 months, and of TN being completely controlled in 22 cases (25%) (Table 1).More than 90% patients with effective treatment reported that they were “much improved” or “very much improved” each month (Table 2).

**Table 1:** Comparison of the prevalence of effective treatment at different time points

**Time (months)**	**Effective, % (no.)**	**Significantly effective, % (no.)**	**Completely controlled, % (no.)**	**Total effective value, % (no.)**
1	31.8 (28)	18.2 (16)	42.0 (37)	92.0 (81)
2	34.1 (30)	19.3 (17)	46.6 (41)	100 (88)
3	19.3 (17)	14.8 (13)	52.3 (46)	86.4 (76)
4	9.1 (8)	13.6 (12)	50.0 (44)	72.7 (64)
5	8.0 (7)	12.5 (11)	50.0 (44)	70.5 (62)
6	8.0 (7)	9.1 (8)	46.6 (41)	63.6 (56)
7	6.8 (6)	9.1 (8)	42.0 (37)	58.0 (51)
8	5.7 (5)	10.2 (9)	37.5 (33)	53.4 (47)
9	6.8 (6)	8.0 (7)	37.5 (33)	52.3 (46)
10	3.4 (3)	9.1 (8)	37.5 (33)	50.0 (44)
11	2.3 (2)	9.1 (8)	36.4 (32)	47.7 (42)
12	3.4 (3)	9.1 (8)	31.8 (28)	44.3 (39)
13	6.8 (6)	8.0 (7)	25.0 (22)	39.8 (35)
14	8.0 (7)	5.7 (5)	25.0 (22)	38.6 (34)

**Table 2:** Comparison of the Patient Global Impression of Change (PGIC) in Patients with a reduction in the VAS score of ≥50% at different time points

**Time (months)**	**Patients with a reduction in the VAS score of ≥50% (n)**	**Very much improved (n**_**1**_**)**	**Much improved (n**_**2**_**)**	**n**_**1+**_**n**_**2**_**/n × 100%**
1	81	45	32	95.06%
2	88	51	32	93.18%
3	78	60	13	93.59%
4	64	56	6	96.88%
5	62	53	8	98.39%
6	56	46	9	96.43%
7	51	42	6	94.12%
8	48	40	7	97.92%
9	46	39	5	95.66%
10	44	40	3	97.73%
11	42	39	2	97.62%
12	39	36	1	94.87%
13	35	30	3	94.28%
14	34	28	6	100%

### Comparison of effective values and pain attack frequency at different doses

There was no significant difference in the prevalence of effective treatment between different dose groups at identical time points between 1 month and 14 months (p > 0.05) (Table 3).

**Table 3:** Comparison of the prevalence of effective treatment at different doses

**Time (months)**	**≤50 U (n = 43)**	**50-100 U (n = 32)**	**≥ 100 U (n = 13)**	**p**
	**Effective value, % (n)**	**Effective value, % (n)**	**Effective value, % (n)**	
1	88.4 (38)	93.8 (30)	100.0 (13)	0.360
2	100.0 (43)	100.0 (32)	100.0 (13)	___
3	88.4 (38)	87.5 (28)	76.9 (10)	0.558
4	76.7 (33)	65.6 (21)	76.9 (10)	0.528
5	74.4 (32)	62.5 (20)	76.9 (10)	0.459
6	72.1 (31)	56.3 (18)	53.8 (7)	0.270
7	65.1 (28)	53.1 (17)	46.2 (6)	0.376
8	60.5 (26)	50.0 (16)	38.5 (5)	0.337
9	58.1 (25)	50.0 (16)	38.5 (5)	0.437
10	58.1 (25)	46.9 (15)	30.8 (4)	0.203
11	55.8 (24)	46.9 (15)	23.1 (3)	0.116
12	51.2 (22)	43.8 (14)	23.1 (3)	0.202
13	48.8 (21)	37.5 (12)	15.4 (2)	0.892
14	46.5 (20)	37.5 (12)	15.4 (2)	0.128

There was no significant difference in the prevalence of pain attack frequency between different dose groups at identical time points between 1 month and 14 months (p > 0.05) (Table 4).

**Table 4:** Comparison of the prevalence of pain attack frequency at different doses

**Time (months)**	**≤50 U (n = 43)**	**50-100 U (n = 32)**	**≥ 100 U (n = 13)**	**p**
	**Attack frequency, time/24 h**	**Attack frequency, time/24 h**	**Attack frequency, time/24 h**	
Before treatment	45.02 ± 85.56	30.40 ± 49.93	83.23 ± 119.49	0.872
1	4.72 ± 10.05	2.56 ± 4.58	1.92 ± 2.96	0.154
2	4.16 ± 9.49	1.63 ± 2.65	2.38 ± 2.87	0.122
3	2.15 ± 4.07	1.31 ± 2.39	1.42 ± 1.78	0.613
4	1.00 ± 2.14	1.43 ± 2.69	1.50 ± 1.90	0.528
5	1.00 ± 2.17	1.50 ± 2.74	1.40 ± 2.12	0.747
6	0.97 ± 2.15	1.33 ± 2.61	1.43 ± 2.44	0.908
7	0.54 ± 1.04	1.59 ± 2.92	2.5 ± 4.18	0.611
8	0.56 ± 1.05	1.50 ± 2.78	1.00 ± 2.24	0.735
9	0.52 ± 0.96	1.43 ± 2.76	2.00 ± 4.47	0.823
10	0.48 ± 0.96	1.00 ± 1.60	0.00 ± 0.00	0.338
11	0.46 ± 0.98	1.00 ± 1.60	0.00 ± 0.00	0.362
12	0.55 ± 1.01	0.71 ± 1.20	0.00. ± 0.00	0.570
13	0.62 ± 1.12	0.83 ± 1.27	2.00 ± 2.83	0.573
14	0.45 ± 0.89	0.75 ± 1.22	1.00 ± 1.41	0.663

### Doses for patients in whom treatment was effective only at 3 months

The number of patients in whom treatment was effective only at 3 months decreased from 88 to 76. The 12 patients in whom treatment was effective only for 3 months comprised 4 cases receiving 25 U, 3 cases receiving 50 U, 2 cases receiving 75 U, 2 cases receiving 100 U, and 1 case receiving 125 U.

### Side effects

Systemic side effects were not observed. Local swelling at injection sites occurred in 3 patients and disappeared within 7 days, 1 cases receiving 25 U, 1 cases receiving 75 U, 1 cases receiving 125 U. Muscle relaxation at injection sites occurred in 10 patients and disappeared within 6–8 weeks, 1 case receiving 45 U, 2 case receiving 50 U, 1 case receiving 70 U, 2 case receiving 75 U, 2 case receiving 100 U, 1 case receiving 120 U, 1 case receiving 125 U. All side effects were mild and disappeared without the need for further treatment.

## Discussion

BTX-A has been used against neuropathic pain, including post-herpetic neuralgia [10], diabetic neuropathic pain [11], occipital neuralgia [12], TN [13] and chronic facial pain [14]. The present study compared the effects of 75 U BTX-A and placebo in the treatment of primary TN for 8 weeks. BTX-A significantly decreased the VAS score 1 week after BTX-A injection and the effect continued for 8 weeks, suggesting that multiple subcutaneous injections of BTX-A in the TN area can significantly relieve pain [5]. Studies have suggested that injection of BTX-A in an area near the zygomatic arch or masseter muscle can also be effective [15,16].

The present study demonstrated that treatment was effective within 4 weeks in 81 cases, and within 4–8 weeks in 7 cases. These findings suggested that observation of ≥8 weeks is necessary for evaluation of therapeutic effects. Five cases showed a decreased VAS score after 2 months and that their pain was completely controlled at 3 months. These results suggest that the therapeutic effect in some cases is slow. Micheli et al. reported that recurrent facial spasm is accompanied simultaneously by recurrent pain after 12 weeks in patients with facial spasms complicated by TN [4]. Therefore, Micheli et al. proposed that the duration of the effect of BTX-A for treating TN is consistent with its relaxation of muscle tension. In general, relaxation of muscle tension by BTX-A can last for 3–6 months. In the present study, the prevalence of effective treatment at 6 months and 14 months was 63.6% and 38.6%, including 41 cases and 22 cases of completely controlled pain, respectively. In 2012, we reported 1 case of TN without recurrence in 2 years [17], which is beyond the period of relaxation of muscle tension elicited by BTX-A. This finding suggests that BTX-A can relieve pain for longer than it can relax muscle tension. Therefore, we think that the analgesic effect of BTX-A is not related to the inhibition of acetylcholine release in nerve–muscle junctions.

With respect to the dose range, reports have suggested values from 12.5 U to 100 U [9], with the range of 50–100 U seeming to be the best. The dose range used in the present study was 25–170 U. The patient mentioned above who remained free of TN for 2 years was the first patient with TN of the third branch that we treated early. The VAS score was 10 and the dose of BTX-A was 170 U (which was the maximum dose we used in all TN patients, she received an additional dose 10 days after the first injection.). At 4 weeks, the VAS score was 0 and, unexpectedly, there was no pain for 3 years.

The present study suggests that there is no significant difference in the therapeutic effect and pain attack frequency between different dose groups at identical time points between 1 month and 14 months. We also showed that effective treatment was observed within 1 month in 81 cases and at 2 months in 88 cases. Effective treatment at 3 months was observed in only 76 cases. That is, therapeutic effect was maintained only for 3 months in 12 patients. The 12 cases showing a therapeutic effect only for 3 months were those using all doses of BTX-A. Patients with a short-term therapeutic effect were mainly those showing a decrease in the VAS score after the first injection. Patients in whom symptoms were completely controlled showed this effect at 3 months (46 cases), of which 14 cases showed no pain at 14 months. These results suggest that the factor influencing the long-term effect of BTX-A is not dose but the extent of the reduction of the VAS score. That is, the more the VAS score decreases, the longer the therapeutic effect is maintained. Therefore, to reach complete control of symptoms and limit the minimal effective dose, we recommend a small dose for the first injection and increase the dose after 2–4 weeks for those in whom complete control of symptoms after the first injection is not observed.

The mechanism of action of BTX-A in pain therapy is not clear. Recent studies suggest that BTX-A can relieve pain through inhibition of the release of cytokines from sensory terminals and by reducing neurogenic inflammation [17]. This effect can be accomplished selectively through inhibition of C-fibers and the transient receptor potential vanilloid (TRPV)1 receptor [18], inhibition of calcitonin gene-related peptide from trigeminal sensory neurons in the brainstem [19], or decrease of peripheral sensitization and central sensitization [20]. In addition, the proposal regarding the existence of BTX-A-sensitive and -insensitive C-fibers and the TRPV1 receptor [18] may explain the different effects observed in different patients.

We observed only mild local side effects. Local swelling at injection sites occurred in 3 patients and disappeared within 7 days (1 cases receiving 25 U, 1 cases receiving 75 U, 1cases receiving 125 U). Muscle relaxation and facial asymmetry at injection sites occurred in 10 patients and disappeared within 6–8 weeks (1 case receiving 45 U, 2 case receiving 50 U, 1 case receiving 70 U, 2 case receiving 75 U, 2 case receiving 100 U, 1 case receiving 120 U, 1 case receiving 125 U), which may have been due to the diffusion of BTX-A into deeper layers of facial muscles as well as the induction of transient muscle paralysis. All side effects were mild and disappeared without the requirement for special treatment.

During the study, we observed more than 90% patients with effective treatment reported that they were “much improved” or “very much improved” each month. That means the treatment not only improved the TN symptoms, but also showed an improvement in the quality of life, emotional function, side effect burden. So, we think the subcutaneous injection of BTX-A for treating TN is a clinical trial achieved a good satisfaction out of participants’ aggregation all of the components of their experience—pain relief, improvement in physical and emotional functioning, side effects and so on.

And for those patients who have lost the injection efficacy, 10 patients choose the surgical treatment, 4 patients received the medical treatment, and 40 patients received re-injection.

## Conclusion

In conclusion, we found that local subcutaneous injection of BTX-A for treating TN had considerable beneficial effects lasting for several months and was safe to use for this indication. One-quarter of patients could reach complete control of pain for >14 months, and maintenance of the therapeutic effect was related to a reduction in the extent of the VAS score after the first injection.

## Competing interests

The authors declare that they have no competing interest.

## Authors’ contributions

YJL study concept and design. SL acquisition of data. HFZ carried out the treatment and studies, drafted the manuscript. YC participated in the sequence alignment, analysis and interpretation. YQM, CHH, CJW revise the manuscript. NCX, YKZ, YC study supervision. All authors read and approved the final manuscript.
